# Measuring health-related quality of life in adolescents and young adults: Swedish normative data for the SF-36 and the HADS, and the influence of age, gender, and method of administration

**DOI:** 10.1186/1477-7525-4-91

**Published:** 2006-12-01

**Authors:** Anna Jörngården, Lena Wettergen, Louise von Essen

**Affiliations:** 1 Department of Public Health and Caring Sciences, Psychosocial Oncology, Uppsala University, Uppsala Science Park, Dag Hammarskjölds väg 10 B, S-751 83 Uppsala, Sweden

## Abstract

**Background:**

There is a paucity of research about health-related quality of life (HRQL) among adolescents, as studies have to a large extent focused on adults. The main aim was to provide information for future studies in this growing field by presenting normative data for the Short Form 36 (SF-36) and the Hospital Anxiety and Depression Scale (HADS) for Swedish adolescents and young adults. Additionally, the influence of age and gender, as well as method of administration, was investigated.

**Methods:**

A sample of 585 persons aged 13–23 was randomly chosen from the general population, and stratified regarding age group (young adolescents: 13–15 years; older adolescents: 16–19 years, and young adults: 20–23 years) and gender (an equal amount of males and females). Within each stratum, the participants were randomized according to two modes of administration, telephone interview and postal questionnaire, and asked to complete the SF-36 and the HADS. Descriptive statistics are presented by survey mode, gender, and age group. A gender comparison was made by independent t-test; and one-way ANOVA was conducted to evaluate age differences.

**Results:**

Effects of age and gender were found: males reported better health-related quality of life than females, and the young adolescents (13–15 years old) reported better HRQL than the two older age groups. The older participants (16–23 years old) reported higher scores when interviewed over the telephone than when they answered a postal questionnaire, a difference which was more marked among females. Interestingly, the 13–15-year-olds did not react to the mode of administration to the same extent.

**Conclusion:**

The importance of taking age, gender, and method of administration into consideration, both when planning studies and when comparing results from different groups, studies, or over time, is stressed.

## Background

Increasingly, the concept "health-related quality of life" (HRQL) has attracted attention in research within the medical and caring sciences [1]. There are many definitions of HRQL, but they can be summarized as referring to a multidimensional psychological construct, which encompasses physical, psychological, social, and functional areas of life, and the impact of health and illness on these aspects [1,2]. In studies, measures of HRQL are often used to evaluate the outcome of different treatments and the relative burden of various diseases [3]. However, when assessing HRQL in diverse clinical groups, it is of utmost importance to have validated normative data for the general population at hand, in order to make meaningful comparisons. The lack of normative data, and well-validated instruments, is an even greater problem when assessing HRQL in adolescents and young adults [4,5]. Historically, these groups have been overlooked to a large extent, as quality of life research has almost exclusively focused on adults [6]. Moreover, studies of general populations that do include a young sample often report data lumped together into large age groups, making it difficult to distinguish information about HRQL in adolescence and young adulthood. Recently, efforts have been made to further develop and validate generic instruments specifically aimed at measuring HRQL in children and adolescents, such as KIDSCREEN and PedsQL [7-9]. Studies show promising results, and add valuable information to the research field. However, it is also useful to be able to include a wider age range in the same sample, avoiding the creation of a gap between the measuring of HRQL in adolescence and adulthood. With this as a starting point, the present study will focus on the Short Form 36 (SF-36) and the Hospital Anxiety and Depression Scale (HADS) used in a young sample (aged 13–23) of the general Swedish population, providing norms and investigating the influence of demographic variables and different methods of administration.

Research has shown that the administration methods influence the way people respond to health status measures. People tend to rate their health and well-being, measured by the SF-36, significantly more favourable in telephone interviews than in questionnaires distributed by mail [10,11]. One explanation for this finding is the difference in perceived anonymity between the two methods [10]. Given this, there is a need for normative data for the two most common modes of administration – telephone interview and postal questionnaire – respectively, in order to provide trustworthy comparison data for future studies.

Additionally, demographic variables such as age and gender have been reported as being of importance, both for HRQL in general [12], and for the specific instruments being examined, and will therefore be taken into consideration in the present study. Lower self-evaluated HRQL as measured by the SF-36 is associated with both increasing age [13,14], and being female [13]. Regarding the age effect, the decline is more marked in the scales reflecting physical health, but has also been detected in the mental health scales [13]. The fact that females report lower scores on the SF-36 has also been replicated in one of the few studies on adolescents [15]. Regarding the HADS, a multitude of studies demonstrate that women score higher on the anxiety subscale, while gender differences are generally not demonstrated in the depression subscale [16]. However, in a study of adolescents by White et al., gender differences exist for both subscales, but there are no significant effects due to age [17].

Regarding the SF-36, normative values for the Swedish population are in existence, reported in the Swedish SF-36 Manual and Interpretation Guide [18]. The youngest group included was aged 15–24 (n = 1451, response rate roughly 70%, 50% females and 50% males). Most of the participants came from a small town (Skellefteå). However, these norms were obtained by postal questionnaire only, and may therefore not be suitable for comparison in studies using telephone interviews. Furthermore, no values are presented for early adolescence (13–14-year-olds). An additional reason for obtaining new values is that almost 15 years have passed since these population studies were performed, implying that the values could have changed.

No previous study has investigated the effect of method of administration, age, and gender on how adolescents and young adults rate HRQL, anxiety, and depression. The main aim of this study is to provide normative data for the SF-36 and the HADS for adolescents and young adults in the Swedish general population (aged 13–23), taking these three factors into consideration. Norms will be presented by method of administration – telephone interview and postal questionnaire – and the potential effect of survey mode will be examined. Additionally, whether gender and age influence the scores on the SF-36 and the HADS will be explored, by survey mode.

## Methods

### Participants

The sample was randomly chosen by Statistics Sweden from their register of the total population. The target population included all individuals covered by civil registration between 13 and 23 years of age, living in three public healthcare regions in Sweden: South, Uppsala/Örebro (Middle) and North. These areas were chosen in order to ensure proper representation by including participants from geographically diverse areas. The sample was stratified with proportional allocation regarding gender (an equal amount of males and females), age group (young adolescents: 13–15 years; older adolescents: 16–19 years; and young adults: 20–23 years), and public healthcare region. The stratification regarding geographical area was included to be able to explore any possible occurrences of systematic differences due to region. This was tested by one-way ANOVA, which did not indicate such differences. Within each stratum the participants were randomized according to the two methods of administration, telephone interview and postal questionnaire. The selected eligible sample consisted of 840 individuals who were invited to participate, 391 of whom were approached through telephone interview and 449 by a postal questionnaire.

### Questionnaires

#### The Short Form 36 (version 1.0)

The SF-36 was developed by an American research group, led by John Ware [19]. It was designed to provide an instrument for the self-evaluation of HRQL which summarized the essence of conceptions of health. Efforts were made to ask as few questions as possible without omitting valuable information, with the aim of simplifying participation and improving the cost effectiveness of data collection. The questionnaire consists of 36 items measuring eight dimensions of life quality: Physical Functioning (PF); Role Physical (RP), which refers to role limitations due to physical difficulties; Bodily Pain (BP); General Health (GH); Vitality (VT); Social Functioning (SF); Role Emotional (RE), which refers to role limitations due to emotional difficulties, and Mental Health (MH). In addition, one single item determines perceived differences in state of health over the past year. Verbal response choices vary from two to six.

Based upon the eight scales, two summary scales have been constructed for physical and mental health respectively [20]. The Physical Component Summary (PCS), is primarily a comprehensive measure of PF, RP, BP and GH, whereas the Mental Component Summary (MCS) mainly encompasses VT, SF, RE and MH. However, the two summaries somewhat overlap, and especially the VT, GH, and SF scales have noteworthy correlations with both components.

The SF-36 has been extensively validated in an American context [21,22], and Swedish studies have shown similar results for data completeness, scaling assumptions, reliability, construct validity and criterion validity [13,18,23]. The SF-36 is described as adequate from early adolescence, and the Swedish manual presents normative data for people aged from 15 to 75+ [18]. Notwithstanding, a very limited amount of studies has focused on adolescents, and most of these principally concern clinical groups. One of the few studies that has come to our attention where the SF-36 has been used with the aim to explore adolescents' self-assessment of HRQL is performed by Goodman et al. (1997). This study investigates how gender and social class affect perceptions of health among 16-year-olds [15].

#### The Hospital Anxiety and Depression Scale

The HADS is a brief self-report screening scale, developed by Zigmond and Snaith (1983) [24], to investigate the prevalence of emotional distress among patients at general medical out-patient clinics. One of the main motives for developing the HADS was the perceived need of a brief questionnaire, which was easy to administer, while still being a source of discriminating information about emotional disorders. Therefore, the scale focuses on the two aspects of psychological health which were considered to have the most relevance, i.e., anxiety and depression. To measure these two dimensions, the scale contains two subscales, one for anxiety and one for depression, each consisting of 7 items (range 0–21). To lessen the possible effects of physical illnesses, no items relating to symptoms that might stem from a somatic condition, such as dizziness and loss of appetite, were included.

There are two ways of interpreting the HADS scores; either by comparing an individual's score to normative values obtained from a sample of the general population, or by using cut-off scores that indicate different levels of clinically relevant distress [25]. Snaith and Zigmond have identified three cut-off levels: a score between 8 and 10 indicate a mild case, 11–14 a moderate case, and 15 or above, a severe case [25,26].

The HADS has been used extensively, and is perceived as performing well when studying aspects of disease and quality of life in clinical as well as general populations [16]. One study of the general population, carried out by Lisspers et al. (1997), concerns psychometric data for a Swedish sample [27]. However, of importance for the present study is that the youngest age group included in their sample consisted of 30–39-year-olds.

The HADS was originally developed for people aged between 16 and 65 [24]. Only one study focusing explicitly on using the HADS in a population sample of adolescents has come to our attention, namely that by White et al. (1999), which aimed at validating the HADS for use with adolescents (aged 12–16) [17]. The study concluded that the HADS is reliable, and has adequate sensitivity and specificity, and is therefore useful for screening adolescents.

### Procedure

The study was approved by the regional ethical review board at the Faculty of Medicine, Uppsala University, Uppsala. The data was collected by Statistics Sweden between March and May 2005.

#### Telephone interview

After completing an organized training programme, five interviewers performed the telephone interviews. All potential participants received a letter containing information about the study. For those under 18, a separate information letter was addressed to the parents. Potential participants were contacted over the telephone by one of the interviewers within a week after the information letter was mailed. Provided that the respondent agreed to participate, and that parental consent was obtained for those under 18, a time was either booked for the interview, or in some cases the interview was conducted directly. The interviewer read the two questionnaires (the SF-36 version 1.0 and the HADS) aloud to the respondent, and recorded his or her responses. As a form of compensation, all participants received a cinema ticket by mail.

#### Postal questionnaire

Potential participants received a letter containing the two questionnaires (the SF-36 version 1.0 and the HADS) and information about the study, as well as a stamped and addressed envelope. In order to obtain parental consent, a separate information letter was addressed to the parents if the potential participant was under the age of 18. Those who did not return the questionnaires within two weeks were sent a reminder. Shortly after their participation, all participants received a cinema ticket by mail, as a form of compensation.

### Statistical analyses

All statistical analyses were conducted using the Statistical Package for the Social Sciences (SPSS) version 14.0 (SPSS Inc, Chicago, IL).

The SF-36 was scored by coding raw scores for each question, and recalibrating, summing, and transforming them into a scale from 0 (worst possible HRQL) to 100 (best possible HRQL), following the standard SF-36 scoring algorithms [18]. Two summary scores, a Physical Component Summary (PCS), and a Mental Component Summary (MCS), are standardized to a mean of 50, with a score above 50 representing better than average function and below 50 poorer than average function. Missing values were substituted if half or more of the items within a subscale were responded to; that is, a person-specific mean score was calculated based on the existing answers. When a HADS questionnaire was partially incomplete, the scale values were estimated by assuming that the missing item(s) had a value equal to the average of those in existence, provided that no more than three items of a subscale were missing.

Sample characteristics are presented descriptively (frequencies and percentages). In order to provide normative data for the SF-36 and the HADS, score distributions are demonstrated. Apart from means and standard deviations, medians, and 1^st ^and 3^rd ^quartiles are presented, as scores were not normally distributed. Regarding the HADS, those percentages of the sample classified as being mild, moderate or severe cases, as indicated by Snaith and Zigmond's criteria [26], are also presented. All of these descriptive analyses were computed by method of administration. Cronbach's alpha coefficients were calculated to estimate the internal consistency reliability of each SF-36 and HADS subscale. According to the generally accepted standard, the alpha coefficient should not fall below 0.70 [28]. In order to make a gender comparison, independent t-test was calculated for the two modes of administration respectively. To evaluate the differences between the three age groups, one-way ANOVA was conducted, followed by the Tukey HSD post-hoc test. Again, the statistical analysis was performed by the two survey modes respectively. P-values ≤ .05 were considered statistically significant.

## Results

### Sample characteristics

Sample demographic characteristics and the response rate are presented in Table 1. The overall response rate was 69.6% (telephone interview 76.7% and postal questionnaire 63.5%). Only 0.8 % of the addresses of those selected for the telephone interview, and 0.7 % of those selected for the postal questionnaire were impossible to find. It was only possible to record the reasons for non-participation among those randomized for the telephone interview. These reasons were: hindered from participating due to illness or language difficulties (2.1%), impossible to reach (9.2%), and not wishing to participate (11.3%). Teenagers whose parents did not give their consent were included as not wanting to participate (n = 7).

**Table 1:** Sample demographic characteristics and response rate, by method of administration and in total

	**Telephone**		**Mail**		**Total**	
	N	%	N	%	N	%
Approached	391	-	449	-	840	-
Response rate	-	76.7	-	63.5%	-	69.6
Participated	300	-	285	-	585	-
Gender						
Male	147	49	130	45.6	277	47.4
Female	153	51	155	54.4	308	52.6
Age						
13–15	89	29.7	91	31.9	180	30.8
16–19	119	39.7	110	38.6	229	39.1
20–23	92	30.7	84	29.5	176	30.1
Region						
South	98	32.7	99	34.7	197	33.7
Uppsala/Örebro	138	46	126	44.2	264	45.1
North	64	21.3	60	21.1	124	21.2

### Normative data for the SF-36 and the HADS for the general Swedish population ages 13–23

Important features of score distributions and estimates of reliability are presented for the HADS in Table 2, and for the SF-36 in Table 3, divided by method of administration. Distributions were skewed, with more subjects giving favourable response choices. The median exceeded the mean for all SF-36 scales, and fell below the mean for the two HADS scales, as expected for relatively healthy general populations. Cronbach's alpha exceeded .70 in all the scales measured by the postal questionnaire, but in the telephone interview mode, three subscales fell below α .70; namely Bodily Pain and Social Functioning in the SF-36, and Depression in the HADS.

**Table 2:** Descriptive statistics, Cronbach's α, and score distributions for the HADS, by method of administration

	**Anxiety**		**Depression**	
	Telephone (n = 300)	Mail (n = 280)	Telephone (n = 300)	Mail (n = 285)
Mean	4.66	5.31	2.52	2.96
SD	3.35	4.14	2.27	3.14
α	.75	.83	.54	.78
Quartile 1	2.00	2.00	1.00	1.00
Median	4.00	5.00	2.00	2.00
Quartile 3	6.00	8.00	3.00	4.00
Range	0–19	0–20	0–14	0–18
% Ceiling	0.0	0.0	0.0	0.0
% Floor	6.0	10.4	14.3	19.3
% Case				
Mild (8–10)	10.7	16.1	2.3	3.9
Moderate (11–14)	4.7	9.1	1.7	2.1
Severe (≥15)	1.7	2.5	0.0	1.4

**Table 3:** Descriptive statistics, Cronbach's α, and score distributions for the SF-36, by method of administration.

**Telephone** **(n = 300)**	**PF**	**RP**	**BP**	**GH**	**VT**	**SF**	**RE**	**MH**	**PCS**	**MCS**
Mean	97.9	89.9	85.4	82.6	69.4	93.3	86.7	80.7	54.7	48.7
SD	7.8	22.8	18.6	15.9	18.5	14.2	27.7	15.0	5.0	9.2
α	.86	.75	.62	.71	.81	.67	.75	.82	-	-
Quartile 1	100	100	72	75	60	87.5	100	76	52.6	45.4
Median	100	100	100	87	75	100	100	84	55.7	51.3
Quartile 3	100	100	100	95	85	100	100	92	57.7	54.9
Range	0–100	0–100	22–100	15–100	10–100	0–100	0–100	20–100	31.2–69.4	5.1–64.8
% Ceiling	82.7	78.7	54.0	13.0	2.7	72.3	77.7	5.7	-	-
% Floor	0.3	2.3	0.0	0.0	0.0	0.3	5.0	0.0	-	-

**Mail** **(n = 277–285)**	**PF**	**RP**	**BP**	**GH**	**VT**	**SF**	**RE**	**MH**	**PCS**	**MCS**

Mean	94.8	86.6	81.6	79.6	64.3	88.2	81.1	76.3	53.7	46.2
SD	13.7	25.9	21.1	18.7	21.6	18.9	32.5	18.9	6.6	11.8
α	.91	.77	.79	.80	.84	.84	.78	.85	-	-
Quartile 1	95	75	72	67	50	75	66.7	68	50.7	42.5
Median	100	100	84	82	66	100	100	80	55.2	50.4
Quartile 3	100	100	100	97	80	100	100	92	57.7	54.1
Range	10–100	0–100	10–100	20–100	0–100	0–100	0–100	16–100	27.7–69.4	2.7–64.8
% Ceiling	69.5	71.6	42.8	18.2	3.5	57.5	69.5	9.1	-	-
% Floor	0.0	3.9	0.0	0.0	0.4	0.4	8.4	0.0	-	-

### Comparison regarding gender

In both methods of administration, males and females differed from each other regarding the scores of the SF-36 and the HADS on a number of subscales, see Table 4. In the telephone interview, significant differences could be detected in five of the SF-36 scales and in the HADS Anxiety scale. Correspondingly, in the mail survey, the same differences were detected with the addition of Bodily Pain and Social Functioning. All the differences were due to the males reporting higher HRQL as measured by the SF-36, and lower levels of symptoms of anxiety as measured by the HADS.

**Table 4:** SF-36 scores and HADS scores by method of administration and gender

**SF-36 and HADS scales**	**Telephone M (SD)**		**Mail M (SD)**	
	Male n = 147	Female n = 153	Male n = 125–130	Female n = 152–155
Physical functioning	98.0 (9.5)	97.7 (5.7)	95.4 (13.7)	94.3 (13.8)
Role-Physical	91.8 (19.0)	88.0 (25.8)	86.6 (26.9)	86.6 (25.1)
Bodily Pain	86.7 (17.6)	84.2 (19.4)	84.4 (19.9)	79.4 (21.8)*
General Health	84.9 (14.3)	80.5 (17.2)*	83.6 (17.0)	76.3 (19.3)***
Vitality	74.0 (17.2)	65.0 (18.6)***	68.5 (19.1)	60.7 (22.9)**
Social Functioning	94.5 (14.7)	92.2 (13.6)	93.0 (13.0)	84.2 (21.9)***
Role-Emotional	89.9 (24.9)	83.6 (29.9)*	90.4 (24.7)	73.3 (36.1)***
Mental Health	83.7 (13.8)	77.9 (15.6)***	81.2 (15.0)	72.1 (20.8)***
Physical Component Summary	54.9 (4.6)	54.6 (5.4)	53.5 (6.5)	53.9 (6.7)
Mental Component Summary	50.5 (8.7)	47.1 (9.4)***	49.7 (8.4)	43.4 (13.3)***
Anxiety	3.82 (2.88)	5.48 (3.57)***	4.17 (3.51)	6.27 (4.39)***
Depression	2.53 (2.07)	2.51 (2.46)	2.85 (2.82)	3.06 (3.39)

* p < .05; ** p = .01; *** p = .001

### Comparison regarding age group

No significant differences were detected between the group of older adolescents (16–19 years old) and the group of young adults (20–23 years old). However, the group of young adolescents (13–15 years old) differed significantly from the two other groups on a number of subscales, in both methods of administration, see Table 5. In the telephone interview mode, there were differences in four SF-36 scales. With reference to the postal questionnaire, the differences were more numerous regarding the SF-36, and both of the HADS scales also differed. All differences derived from the fact that the youngest age group reported higher HRQL as measured by the SF-36, and lower levels of symptoms of emotional distress as measured by the HADS. However, it should be noted, that in the telephone interview mode, the youngest age group (13–15 years) only differed from the 20–23-year-olds and not from the 16–19-year-olds in Social Functioning and Mental Component Summary, and differed only from the 16–19-year-olds and not from the 20–23-year-olds regarding Role-Emotional subscale scores.

**Table 5:** SF-36 scores and HADS scores by method of administration and age group

**SF-36 and HADS scales**	**Telephone M (SD)**				**Mail M (SD)**			
	13–15 n = 89	16–19 n = 119	20–23 n = 92	F df = 2	13–15 n = 88–91	16–19 n = 106–110	20–23 n = 83–84	F df = 2
Physical Functioning	98.9 (3.6)	97.5 (10.0)	97.2 (7.5)	1.27	93.4 (18.4)	94.2 (12.8)	96.8 (7.7)	1.37
Role-Physical	92.4 (19.0)	88.7 (23.2)	89.1 (25.5)	0.77	90.1 (22.3)	83.3 (28.6)	87.2 (25.6)	1.69
Bodily Pain	87.8 (17.9)	84.5 (18.5)	84.4 (19.3)	1.06	89.9 (14.5)	78.2 (22.6)	77.4 (22.6)	10.75***
General Health	84.7 (14.3)	81.4 (17.1)	82.2 (15.7)	1.16	89.2 (12.5)	75.0 (21.0)	75.4 (17.0)	19.64***
Vitality	74.3 (14.8)	67.9 (20.0)	66.6 (18.9)	4.68**	76.4 (16.7)	58.7 (22.0)	58.8 (20.5)	23.96***
Social Functioning	96.5 (9.4)	92.3 (14.7)	91.6 (16.7)	3.26*	95.9 (8.4)	85.0 (19.7)	84.2 (23.1)	11.72***
Role-Emotional	92.9 (22.2)	83.5 (30.0)	85.0 (28.7)	3.25*	91.6 (22.9)	76.7 (35.1)	75.8 (35.2)	7.05***
Mental Health	83.1 (13.2)	80.4 (14.7)	78.9 (16.8)	1.81	84.6 (14.0)	73.6 (20.1)	71.0 (19.0)	14.14***
Physical Component Summary	55.1 (3.7)	54.5 (5.5)	54.6 (5.5)	0.46	54.4 (6.2)	52.9 (7.0)	54.0 (6.6)	1.26
Mental Component Summary	50.9 (7.5)	48.0 (9.3)	47.6 (10.3)	3.55*	51.8 (6.8)	44.2 (12.5)	43.0 (13.2)	16.27***
Anxiety	4.63 (2.85)	4.60 (3.25)	4.78 (3.90)	0.09	3.47 (3.61)	6.14 (4.08)	6.19 (4.17)	14.05***
Depression	2.26 (2.22)	2.59 (1.83)	2.68 (2.78)	0.89	1.99 (2.69)	3.34 (3.23)	3.53 (3.26)	6.83***

* p < .05; ** p = .01; *** p = .001

### Normative data for the SF-36 and the HADS by age, gender, and method of administration

As all the three factors age, gender, and method of administration were found to significantly affect the reports of HRQL, anxiety, and depression, normative values are in addition presented divided by the three variables simultaneously, see Table 6. Numerically, the most "best values" within each subscale (reflecting the highest HRQL or the lowest level of emotional distress) are found among the male 13–15-year olds in the mail mode, while the most "worst values" within each subscale (reflecting the lowest HRQL or the highest level of emotional distress) are reported by the female 20–23-year olds in the mail mode.

**Table 6:** SF-36 scores and HADS scores by age, gender, and method of administration

**SF-36 and HADS scales**	**Telephone M (SD)**			**Mail M (SD)**		
	13–15 n = 89	16–19 n = 119	20–23 n = 92	13–15 n = 88–91	16–19 n = 106–110	20–23 n = 83–84
Physical Functioning						
Male	99.4 (2.2)	97.6 (13.3)	97.2 (7.9)	93.6 (17.2)	95.7 (13.4)	96.9 (8.1)
Female	98.4 (4.5)	97.5 (5.4)	97.3 (7.1)	93.3 (19.7)	93.1 (12.3)	96.7 (7.6)
Role-Physical						
Male	93.8 (16.3)	91.2 (19.8)	90.8 (20.7)	89.8 (23.7)	81.4 (29.2)	89.6 (27.0)
Female	91.1 (21.4)	86.3 (25.9)	87.5 (29.7)	90.4 (21.0)	84.8 (28.2)	85.4 (24.6)
Bodily Pain						
Male	86.9 (18.3)	87.0 (18.1)	86.3 (16.7)	93.5 (14.0)	77.9 (20.7)	81.9 (21.1)
Female	88.8 (17.7)	82.1 (18.6)	82.4 (21.6)	86.4 (14.2)	78.4 (24.1)	74.0 (23.3)
General Health						
Male	86.8 (11.7)	85.1 (14.9)	82.9 (15.3)	92.5 (9.7)	79.8 (19.4)	78.1 (16.6)
Female	82.7 (16.4)	78.0 (18.3)	81.6 (16.2)	86.2 (14.1)	71.1 (21.7)	73.3 (17.2)
Vitality						
Male	78.2 (12.1)	75.5 (18.9)	68.3 (18.0)	78.9 (14.0)	61.2 (22.0)	65.8 (14.6)
Female	70.6 (16.4)	60.0 (18.4)	65.0 (19.7)	74.0 (18.8)	56.6 (21.9)	53.5 (22.8)
Social Functioning						
Male	97.7 (6.2)	93.4 (17.1)	92.6 (17.0)	96.9 (8.1)	90.6 (15.4)	91.7 (13.4)
Female	95.3 (11.7)	91.3 (12.3)	90.5 (16.5)	95.0 (8.6)	80.5 (21.6)	78.6 (27.2)
Role-Emotional						
Male	95.5 (18.5)	88.9 (26.2)	85.9 (28.1)	95.5 (18.5)	86.4 (28.8)	89.8 (25.0)
Female	90.4 (25.2)	78.5 (32.6)	84.1 (29.6)	87.8 (26.2)	68.9 (37.9)	65.3 (38.3)
Mental Health						
Male	86.8 (9.4)	84.1 (14.4)	80.2 (16.1)	87.0 (12.8)	78.9 (15.3)	77.2 (15.4)
Female	79.5 (15.4)	76.9 (14.2)	77.7 (17.6)	82.2 (14.9)	69.4 (22.6)	66.3 (20.2)
Physical Component Summary						
Male	55.0 (2.9)	54.8 (5.5)	54.9 (4.8)	54.6 (6.2)	52.4 (6.1)	53.7 (7.2)
Female	55.3 (4.3)	54.2 (5.5)	54.3 (6.2)	54.1 (6.2)	53.3 (7.6)	54.3 (6.2)
Mental Component Summary						
Male	52.7 (4.5)	50.5 (9.7)	48.3 (10.0)	53.3 (5.1)	47.9 (9.3)	48.0 (9.3)
Female	49.1 (9.2)	45.7 (8.3)	47.0 (10.6)	50.5 (7.9)	41.3 (13.8)	39.1 (14.4)
Anxiety						
Male	3.59 (2.37)	3.79 (3.12)	4.07 (3.04)	2.34 (2.58)	5.17 (3.58)	5.08 (3.60)
Female	5.64 (2.93)	5.34 (3.22)	5.50 (4.52)	4.58 (4.12)	7.00 (4.29)	6.96 (4.42)
Depression						
Male	2.32 (1.91)	2.49 (1.74)	2.78 (2.55)	1.82 (2.29)	3.76 (3.30)	2.89 (2.28)
Female	2.20 (2.51)	2.68 (1.92)	2.59 (3.02)	2.15 (3.04)	3.00 (3.15)	4.02 (3.79)

## Discussion

Normative data for the SF-36 and the HADS have been obtained from a large and stratified sample of the general population of adolescents and young adults in Sweden. It should form an important basis of comparison for future studies of these age groups, both of clinical groups and of the general population.

When assessing HRQL in adolescents, the question is raised whether to use questionnaires specifically designed for children and adolescents or instruments like the SF-36 and the HADS that are mainly developed for adults. Both approaches are entailed with several advantages and disadvantages, some of which are broached by Eiser and Morse in an exhaustive systematic review [29]. It can be questioned whether the domains considered in adult measures are appropriate to determine quality of life in children, and whether they have the same meaning for children as for adults. On the other hand, employing well-known and recognized questionnaires for adults has the benefit of drawing on established knowledge. It also makes it possible to investigate a sample of both adolescents and adults using the same questionnaires, something which is crucial for evaluating changes in quality of life across the life span and longitudinal follow-up.

In line with findings from the overwhelming majority of research about HRQL, the males reported higher scores than the females [5,15,17,30]. The salient differences in the SF-36 were mainly associated with mental and emotional aspects of health, whereas few differences were found in the subscales more closely connected to physical health. Regarding the HADS, females reported a higher degree of anxiety, but no significant differences could be detected in the depression subscale. This is also in accordance with most of the research where the HADS is used [16], but differs from the previously mentioned study on adolescents by White et al. [17], where gender differences were identified in both subscales.

Although the systematic difference in subjective well-being between males and females is a common finding, very few explanations or interpretations have been presented as to why this is the case. One researcher who does focus on this matter is Helen Sweeting, who reviews findings on gender differences in health among children and adolescents [31]. She concludes that girls in no way suffer from worse health than boys during childhood, but that these differences emerge in early-mid adolescence. Furthermore, she calls attention to the role that psychological factors play when it comes to physical illnesses: "It is [...] possible that the excess in physical complaints among females which begins in adolescence arises, at least in part, as a direct result of the relative lowering in their psychological well-being at this age" (p. 88). That attention should be directed towards the psychological factors seems to be a feasible conclusion also of the present study. It may be that girls experience greater, and/or different, stressors than boys do from adolescence and onwards, when the differing cultural expectations of the two genders become more evident. In any event, more research is needed that not only establishes the existence of gender differences in self-evaluated health and well-being, but also further investigates what these differences entail and what consequences they involve.

In some respects, the group of young adolescents (13–15 years old) reported higher HRQL and less emotional distress than the older participants. It appears as if increasing age is also connected to a decrease in HRQL among adolescents and young adults. This too is in agreement with previous research [5,32]. As other researchers have pointed out, these differences make sense and are consistent with the augmented levels of stress that the transition from childhood to adulthood involves [32,33]. It is not surprising that both internal and external stressors associated with this time in life, such as striving for autonomy and forming a mature self-image, as well as forming relationships and achieving at school or professionally, are mirrored in the assessment of HRQL.

A strong point of the present study is that the normative data is presented according to survey mode – telephone interview or postal questionnaire. It is evident that the choice of administration method has a decisive influence on the data that will be obtained. Apart from the statistical difference between the two modes of data collection, a central matter is whether this difference is sufficiently large to have any clinical importance. No minimal clinically important difference standards have been established for the SF-36. However, studies of the score profiles of various clinically defined patient groups have suggested that a 3–5 point change in scale scores may represent a clinically important difference [34-36]. Although this change may be considered small, it could at least partly cloud the impact of an intervention evaluated with an initial interview and a postal follow-up questionnaire, especially since interventions seldom can be expected to be a complete success. Therefore, it is strongly recommended to use the same mode of administration when comparing groups or changes over time.

It is of interest that different groups seem to respond to the mode of administration in diverse ways and to varying degrees, which is also something that researchers should take into account when planning the design of future studies. Both gender and age were shown to have an impact on how people responded to the two methods of administration. Males and females alike tended to report better scores in the telephone interview compared to the postal questionnaire, but the difference was more marked among the females. Contrary to both adults in general and the two older age groups, the youngest age group did not evaluate their HRQL more positively when interviewed over the telephone than when filling in a postal questionnaire. This finding is surprising, and we feel that more research is needed to understand why the youngest respondents react differently to survey mode. One explanation could be that the young adolescents do not follow the rules of social desirability to the same extent, and consequently feel no need to adjust their responses according to expectations.

There are some methodological limitations which should be mentioned. To minimize the risk of possible regional differences, the participants were stratified regarding three geographical areas. However, the regions chosen are vast and encompass small towns and big cities as well as rural areas. Differences due to these circumstances might exist but not come to light in this design. Additionally, there was a difference in response rate between males and females, resulting in more females than males responding to the mail-administered questionnaires. As females generally seem to report worse HRQL, this might have had some influence on the result.

## Conclusion

In conclusion, the study provides pertinent and valuable information about measuring HRQL in adolescents and young adults, a field which has been the subject of little research. In addition, the study presents current normative data for the SF-36 and the HADS for this age group. Furthermore, attention is being directed towards matters that should be investigated further, such as what factors underlie the gender difference which seems to appear in adolescence, and what makes young adolescents respond differently to survey mode used. Most of all, the results stress the importance of taking age, gender, and method of administration into consideration, both when planning studies and when comparing results from different groups, studies, or over time.

## Competing interests

The author(s) declare that they have no competing interests.

## Authors' contributions

AJ wrote the manuscript and conducted the analysis of the data. LW participated in data analysis and revised the manuscript critically. LvE conceived of the design of the study, participated in the analysis of the data and revised the manuscript critically.
